# Rapamycin Attenuates Acute Seizure-induced Astrocyte Injury in Mice in Vivo

**DOI:** 10.1038/s41598-017-03032-0

**Published:** 2017-06-06

**Authors:** Dongjun Guo, Jia Zou, Michael Wong

**Affiliations:** Department of Neurology and the Hope Center for Neurological Disorders, Washington University School of Medicine, St. Louis, MO 63110 USA

## Abstract

Astrocytes have been implicated in epileptogenesis and seizure-induced brain injury. Pathological studies reveal a variety of structural abnormalities in astrocytes, such as vacuolization and astrogliosis. While *in vivo* imaging methods have demonstrated rapid changes in astrocytes under a variety of physiological and pathological conditions, the acute effects of seizures on astrocyte morphology *in vivo* and corresponding mechanisms of seizure-induced astrocytic injury have not been documented. In this study, we utilized *in vivo* two-photon imaging to directly monitor the acute structural effects of kainate-induced seizures on cortical astrocytes. Kainate seizures cause an immediate, but transient, vacuolization of astrocytes, followed over several days by astrogliosis. These effects are prevented by pre- or post-treatment with rapamycin, indicating the mTOR pathway is involved in mediating seizure-induced astrocyte injury. These finding have clinical implications for mechanisms of seizure-induced astrocyte injury and potential therapeutic applications with mTOR inhibitors.

## Introduction

Astrocytes are a group of specialized glial cells in the central nervous system (CNS). Major roles of astrocytes include maintenance of ion and neurotransmitter homeostasis, metabolism, and regulation of synaptic development and signaling. Recent evidence indicates that astrocytes are also involved in epileptogenesis and seizure-related brain injury^1–3^. Pathological studies have documented a variety of abnormalities in astrocytes, such as astrocyte vacuolization, cell death and astrogliosis, in specimens from human and animal models of epilepsy. In particular, astrogliosis is especially common in epilepsy and is characterized by morphological and functional changes in astrocytes, including hypertrophy of primary processes, variable upregulation of glial fibrillary acidic protein (GFAP), and in some cases, increased astrocyte proliferation. Recent advances with *in vivo* imaging have revealed dynamic changes in neurons and glia that were not previously appreciated in pathological studies, including rapid effects of seizures on dendritic spines^4–6^, but the acute effects of seizures on the structure of astrocytes are not well documented. Understanding the changes in astrocytes *in vivo* following seizures could provide the opportunity to clarify the specific mechanistic roles of astrocytes in epilepsy and to develop novel therapeutic approaches to prevent seizures or their consequences.

Astrocytes have been implicated in promoting epileptogenesis via a diversity of mechanisms, such as increased gap junction coupling, impaired glutamate transporter function, and disruption of the blood-brain barrier^2^. Several studies suggest that the mammalian target of rapamycin (mTOR) pathway is activated in astrocytes in some types of epilepsy or in animal models^7, 8^. Other studies show that kainate (KA) induced seizures cause activation of the mTOR pathway and the mTOR inhibitor, rapamycin, prevents this mTOR activation and reduces seizure-induced dendritic injury and subsequent development of epilepsy^6, 9^. Therefore, mTOR inhibitors, such as rapamycin, may also represent a rational and efficacious strategy for preventing astrocyte injury in epilepsy.

In this study, we characterized the rapid, dynamic structural changes in astrocytes *in vivo* following KA-induced seizures utilizing two-photon excitation laser scanning microscopy (2PLSM). We also tested the hypothesis that treatment with rapamycin initiated before or after KA-induced seizures (pretreatment or post-treatment) has protective effects against seizure-induced astrocyte injury.

## Results

### KA-induced seizures cause rapid, dynamic morphological changes in astrocytes

**Figure 1:**
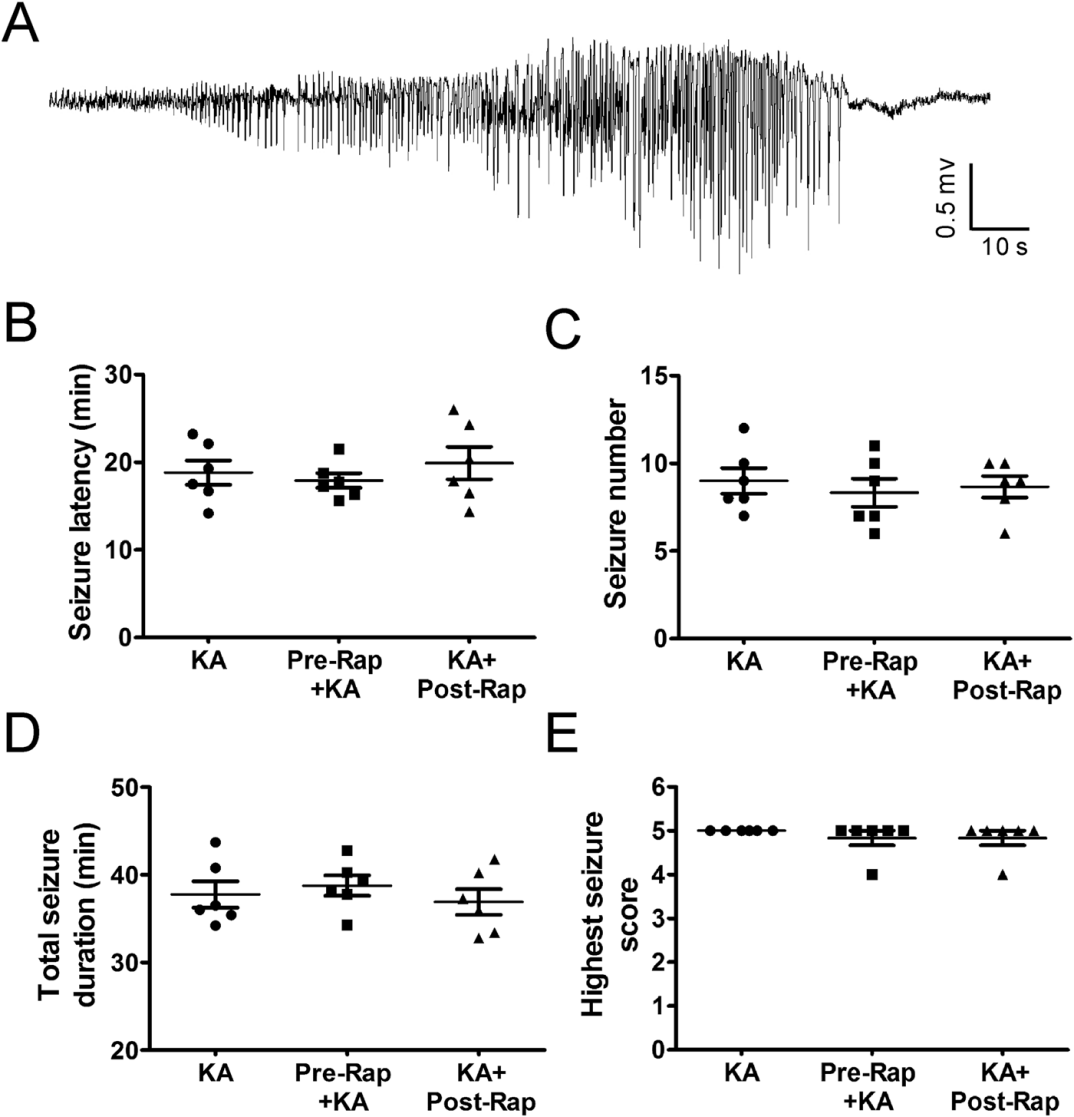
Properties of acute KA-induced status epilepticus and lack of effect of rapamycin pre-treatment. (**A**) Representative electrographic seizure following KA injection. (**B–E**) Rapamycin pre-treatment (6 mg/kg, i.p., 48 hr and 24 hr prior to KA) and post-treatment (6 mg/kg i.p., daily for one week, starting immediately after seizure termination) have no effect on the properties of seizure latency, number, duration, and severity during the acute episode of KA-induced status epilepticus (defined as >30 min of cumulative electrographic seizures). (n = 6 per group; One-way ANOVA with Tukey’s test, p > 0.05).

**Figure 2:**
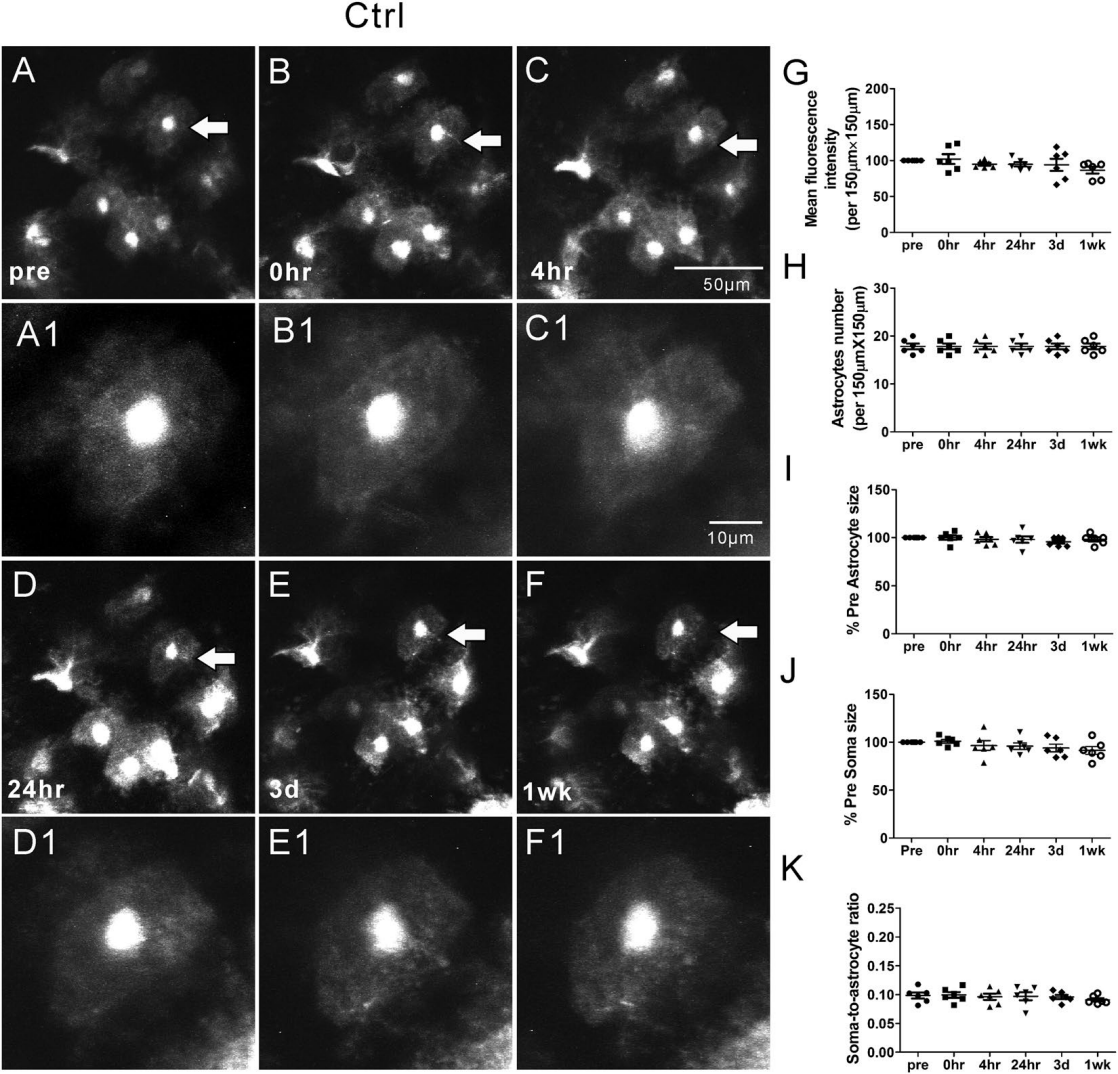
Representative *in vivo* images of astrocytes and quantitative analysis of astrocyte morphology features in the Ctrl group. Under normal physiological conditions, astrocytes typically have a characteristic bushy appearance consisting of thin process (**A**, A1). No obvious astrocytes vacuolization and morphological changes (**B–F**, B1–F1) were observed over a one-week period. No significant changes in mean fluorescence intensity (**G**), astrocyte number (**H**), astrocyte size (**I**), soma size (**J**) and soma-to-astrocyte ratio (**K**) occurred during a one-week period. (n = 6; one-way ANOVA or Kruskal-Wallis test, p > 0.05). The arrows in the lower magnification images indicate the astrocytes displayed in the higher magnification images.

*In vivo* time-lapse 2PLSM has been utilized to examine the rapid and dynamic structural changes in astrocytes in mouse models of stroke and traumatic brain injury^10, 11^. Here, we used a similar strategy to investigate whether astrocytes undergo rapid, dynamic changes immediately following KA-induced seizures and for a week thereafter. Seizures were induced by KA and terminated after 30–45 minutes of cumulative electrographic seizure activity (Fig. 1). First of all, under normal physiological conditions, astrocytes maintained a relatively stable number and morphology including astrocyte size, soma size and soma-to-astrocyte ratio, with a bushy appearance and thin processes throughout the one week observation period in control mice (Ctrl group; Fig. 2). Mean fluorescence intensity (GFAP-driven GFP intensity) also remained stable over time. No obvious astrocyte vacuolization or astrogliosis was observed in control mice (Table 1, Fig. 2A–F).

**Table 1:** Effect of rapamycin treatment on kainate seizure induced astrocyte vacuolization.

Group/Time after seizures	Total Astrocytes	No Vacuolization	Vacuolization
**Ctrl**			
Pre-seizure	107	107 (100%)	0 (0%)
*0 hr	107	107 (100%)	0 (0%)
*4 hr	107	107 (100%)	0 (0%)
*24 hr	107	107 (100%)	0 (0%)
*3 d	107	107 (100%)	0 (0%)
1 wk	107	107 (100%)	0 (0%)
**Rap**			
Pre-seizure			
*0 hr	118	118 (100%)	0 (0%)
*4 hr	118	118 (100%)	0 (0%)
*24 hr	118	118 (100%)	0 (0%)
*3 d	118	118 (100%)	0 (0%)
1 wk	118	118 (100%)	0 (0%)
**KA**			
Pre-seizure	106	106 (100%)	0 (0%)
0 hr	106	26 (24.5%)	80 (75.5%)
4 hr	106	23 (21.7%)	83 (78.3%)
24 hr	106	71 (67.0%)	35 (33.0%)
3 d	106	82 (77.4%)	24 (22.6%)
1 wk	106	106 (100%)	0 (0%)
**Pre-Rap + KA**			
Pre-seizure	107	107 (100%)	0 (0%)
*0 hr	107	103 (96.3%)	4 (3.7%)
*4 hr	107	103 (96.3%)	4 (3.7%)
*24 hr	107	104 (97.2%)	3 (2.8%)
*3 d	107	107 (100%)	0 (0%)
1 wk	107	107 (100%)	0 (0%)
**KA + Post-Rap**			
Pre-seizure	89	89 (100%)	0 (0%)
*0 hr	89	59 (66.3%)	30 (33.7%)
*4 hr	89	60 (67.4%)	29 (32.6%)
24 hr	89	66 (74.2%)	23 (25.8%)
*3 d	89	81 (91.0%)	8 (9.0%)
1 wk	89	89 (100%)	0 (0%)

^*^p < 0.05 vs. KA by Chi-square.

**Figure 3:**
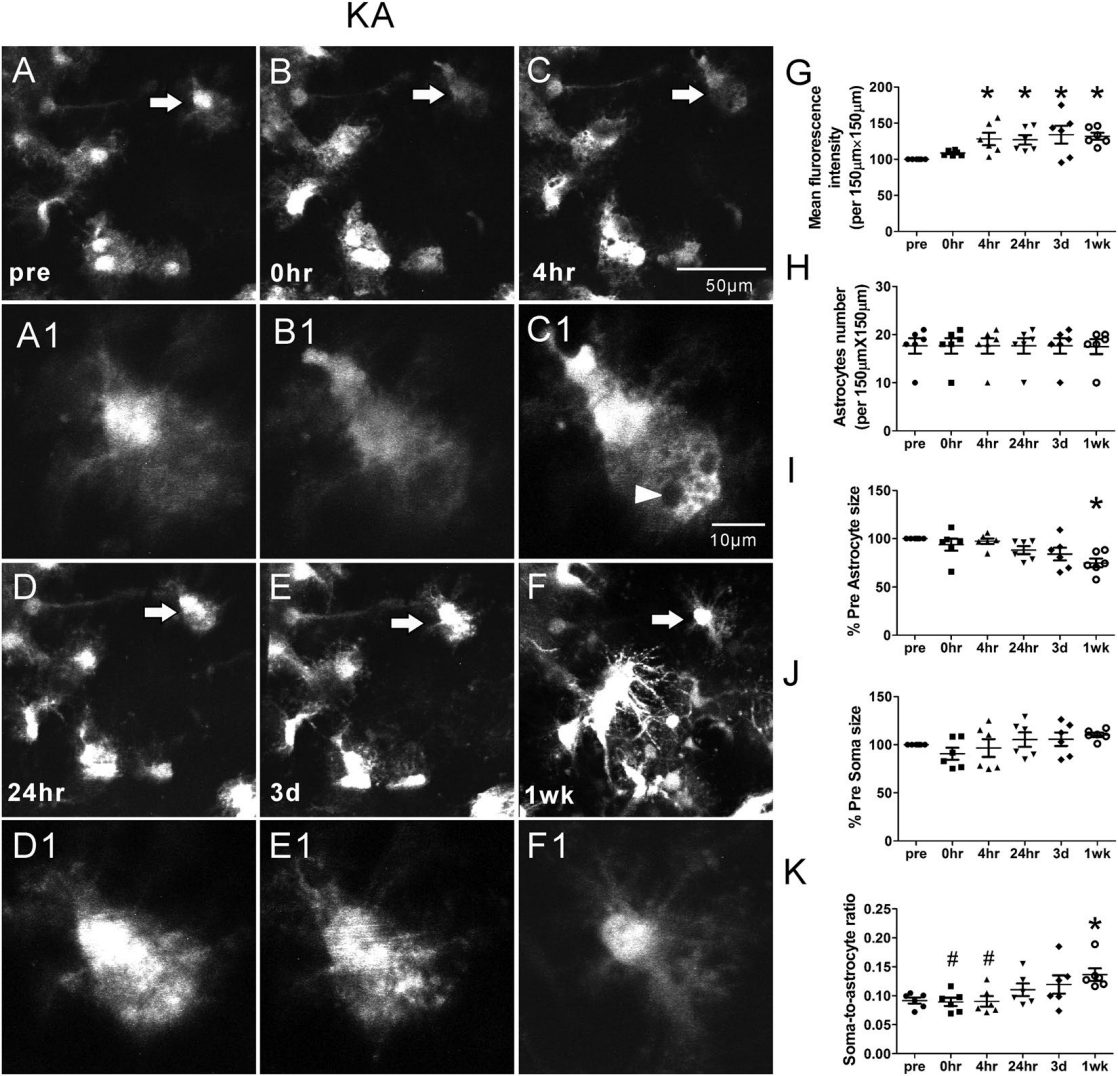
Representative *in vivo* images of astrocytes and quantitative analysis of astrocyte morphology features in the KA group. KA induced seizures caused acute astrocyte injury including vacuolization in most astrocytes followed by astrogliosis (**B-F**, B1–F1). Vacuolization of astrocytes occurred immediately after seizures, peaked at 4 hr and persisted up to 3 days (arrow head in C1). One week after seizures, astrogliosis developed, characterized by a decrease in astrocyte size and loss of their classic bushy appearance, with fine individual processes becoming more prominent and extensive (**F**, F1). No significant proliferation of new astrocytes was observed. However, mean fluorescence intensity increased at 4 hr, 24 hr, 3 d and 1 wk after seizures (**G**). Astrocyte size decreased significantly at 1 wk after seizures (**I**) and the soma-to-astrocyte ratio increased at 1 wk when compared to pre-seizure (**J**) (n = 6; *p < 0.05 compared with pre, by Kruskal-Wallis test with Dunns post-test). The arrows in the lower magnification images indicate the astrocytes displayed in the higher magnification images.

In contrast, KA induced seizures caused acute astrocyte injury characterized initially by vacuolization in ~80% astrocytes, followed by astrogliosis (KA group; Fig. 3, Table 1), which was absent in control mice. Astrocyte vacuolization occurred immediately after seizures, peaked at 4 hr and persisted up to 3 days but then resolved by one week (Fig. 3A–F, Table 1). Between 24 hr to one week after seizures, astrogliosis developed, characterized by an increase in GFAP-driven GFP fluorescence intensity (Fig. 3G; p < 0.05 by Kruskal-Wallis test), decrease in astrocyte size (Fig. 3I; p < 0.05 by Kruskal-Wallis test) and loss of their classic bushy appearance, with fine individual processes becoming more prominent, extensive, and hypertrophied (Fig. 3B–F), without a significant change in astrocyte number (Fig. 3H, p > 0.05). Correspondingly, we did not observe any evidence of death of existing astrocytes or proliferation of new astrocytes during the 1 wk period after seizures, as evident by a lack of change in the number of astrocytes followed individually and serially. The size of the soma also did not change during this period (Fig. 3J, p > 0.05) while the ratio of the soma to astrocyte increased at 1 wk after seizures due to the decrease in astrocyte size (Fig. 3K, p < 0.05 by one-way ANOVA).

### Rapamycin treatment significantly attenuates seizure-induced acute astrocyte injury

**Figure 4:**
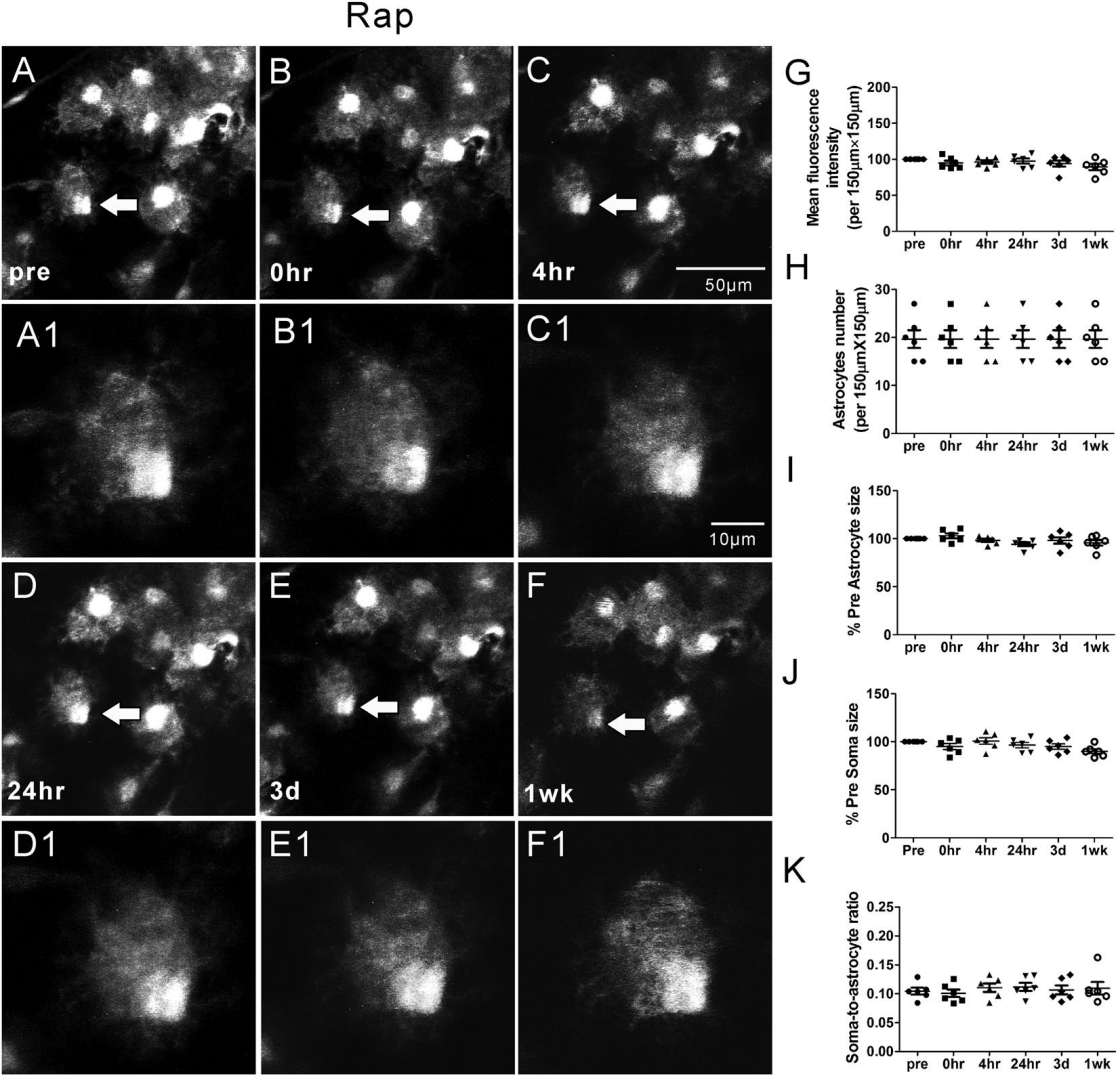
Representative *in vivo* images of astrocytes and quantitative analysis of astrocyte morphology features in the Rap group. Astrocytes typically have a characteristic bushy appearance consisting of thin processes (**A**, A1). No obvious astrocytes vacuolization and morphological changes (**B–F**, B1–F1) were observed over a one-week period. No significant changes in mean fluorescence intensity (**G**), astrocyte number (**H**), astrocyte size (**I**), soma size (**J**) and soma-to-astrocyte ratio (**K**) occurred during a one-week period. (n = 6; one-way ANOVA or Kruskal-Wallis test with Dunns post-test, p > 0.05). The arrows in the lower magnification images indicate the astrocytes displayed in the higher magnification images.

**Figure 5:**
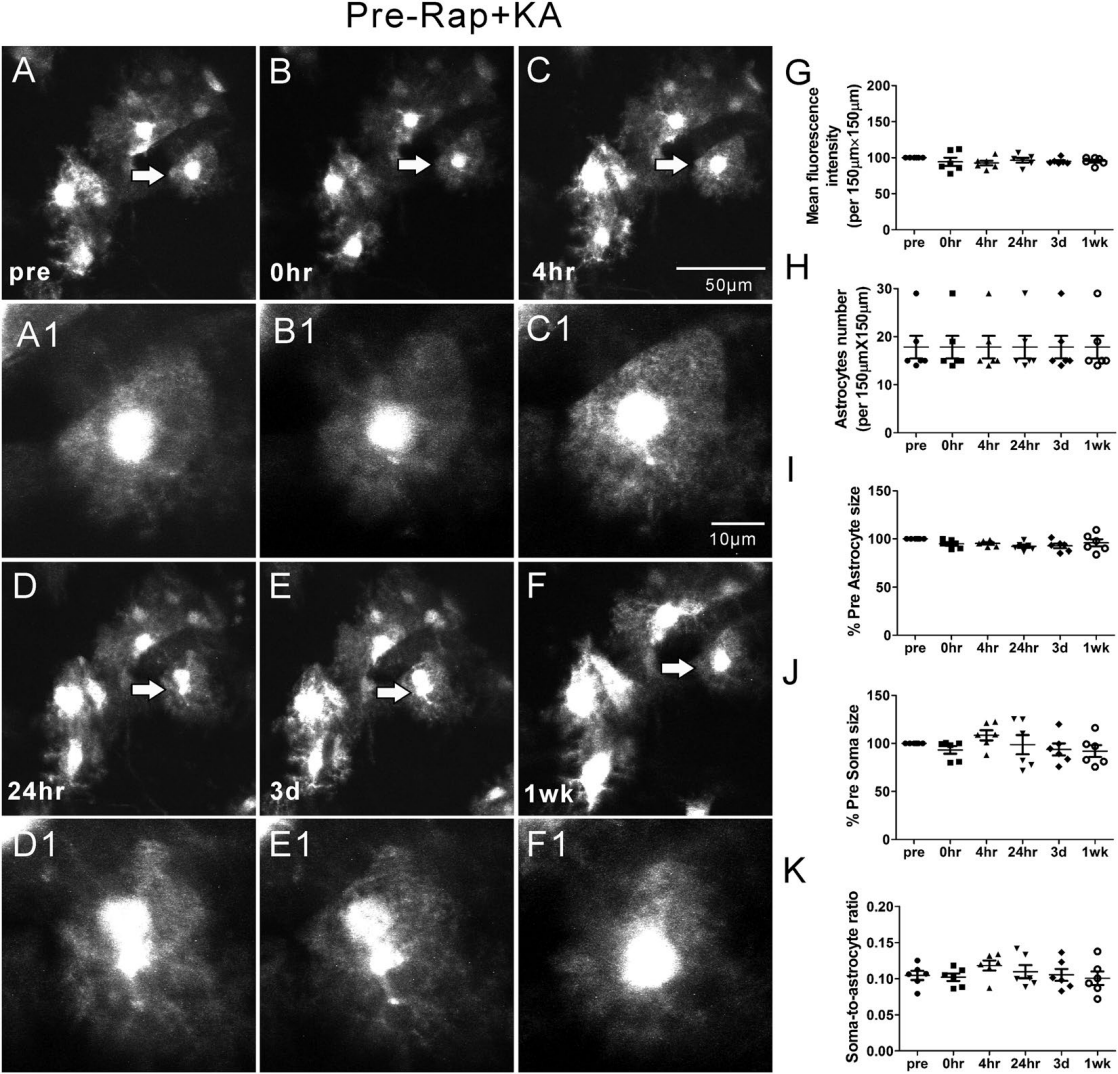
Representative *in vivo* images of astrocytes and quantitative analysis of astrocyte morphology features in the Pre-Rap + KA group. With rapamycin pre-treatment, the astrocytes reserved the normal bushy appearance after KA induced seizures. No obvious morphological change, vacuolization and astrogliosis were observed in most astrocytes (**A–F**, A1–F1). The mean fluorescence intensity, astrocyte number, astrocyte size, soma size and soma-to-astrocyte ratio did not change over a one-week period (**G–K**) (n = 6; one-way ANOVA or Kruskal-Wallis test with Dunns post-test, p > 0.05). The arrows in the lower magnification images indicate the astrocytes displayed in the higher magnification images.

**Figure 6:**
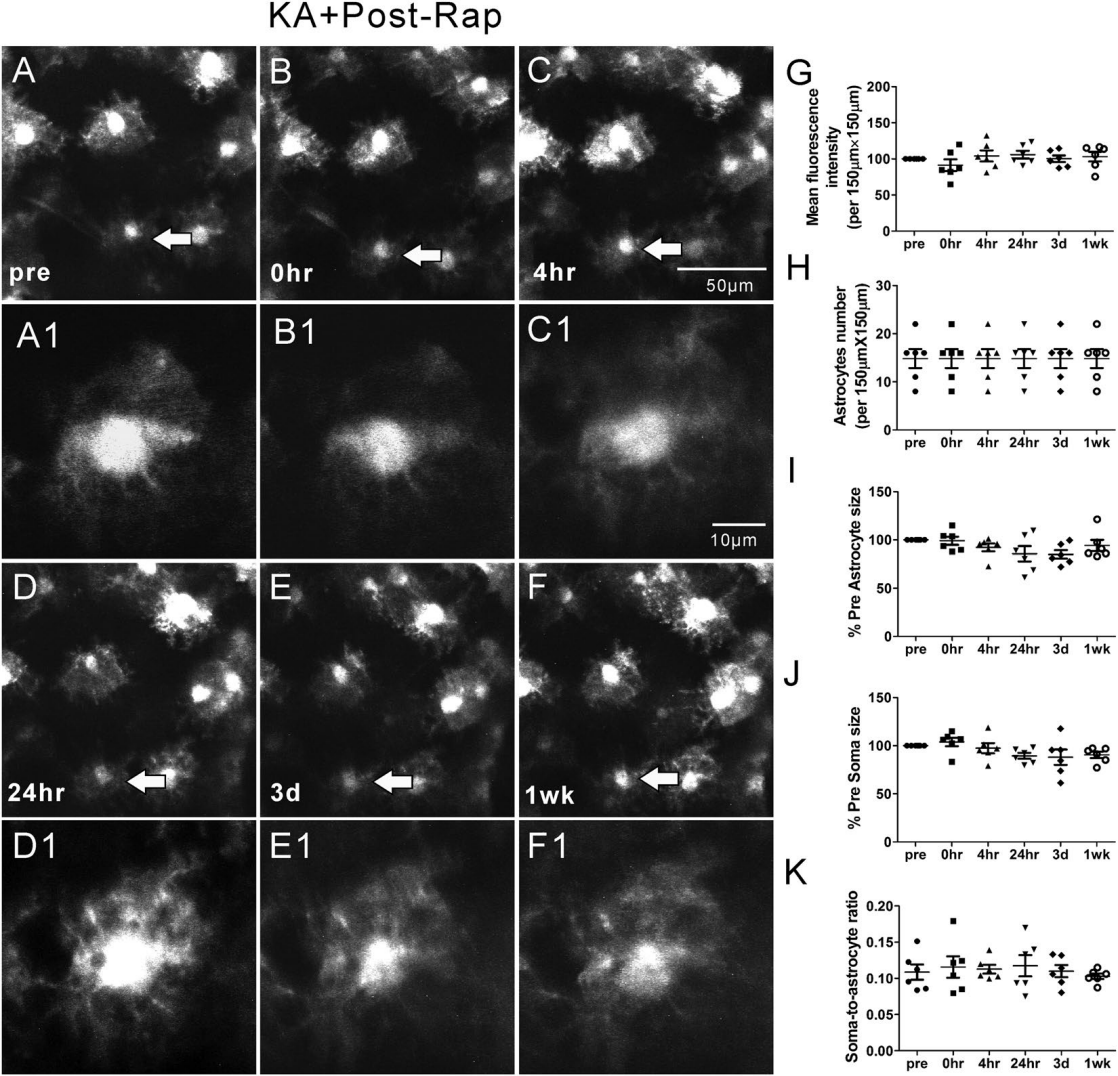
Representative *in vivo* images of astrocytes and quantitative analysis of astrocyte morphology features in the KA + Post-Rap group. With rapamycin post-treatment, most astrocytes present the similar bushy appearance with fine processes as pre-seizure’s condition. No obvious morphological change, vacuolization and astrogliosis were observed in most astrocytes (**A–F**, A1–F1). Compared to pre-seizure condition, mean fluorescence intensity, astrocyte number, astrocyte size, soma size and soma-to-astrocyte ratio did not change over a one-week period (**G–K**) (n = 6; one-way ANOVA or Kruskal-Wallis test with Dunns post-test, p > 0.05). The arrows in the lower magnification images indicate the astrocytes displayed in the higher magnification images.

**Figure 7:**
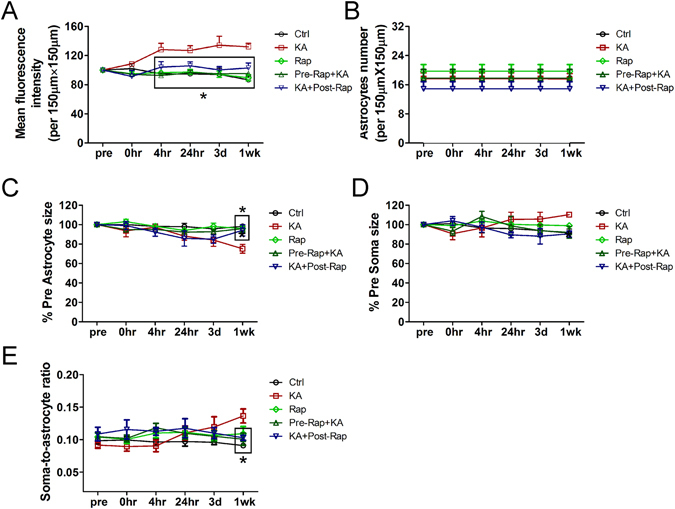
Quantitative analysis of effect of rapamycin treatment on KA seizures induced fluorescence intensity, astrocyte number and morphological changes (astrocyte size, soma size and soma-to-astrocyte ratio) over a one-week period. (**A**) Changes in mean fluorescence intensity over a one-week period in Ctrl, Rap, KA, Pre-Rap + KA, and KA + Post-Rap groups. Starting from 4 hr after KA seizures, mean fluoroscence intensity increased significantly in KA group when compared to Ctrl, Rap, Pre-Rap + KA, and KA + Post-Rap groups. Both rapamycin pre- and post- treatment reversed the increase in fluorescence intensity. (*p < 0.05 compared with KA at 4 hr, 24 hr, 3 d and 1 wk, by repeated measures two-way ANOVA with Bonferroni posttests). (**B**) Changes of astrocyte number over a one-week period in Ctrl, Rap, KA, Pre-Rap + KA, and KA + Post-Rap groups. There is no significant difference among the five groups in astrocytes number at all observed time-points (p > 0.05, by repeated measures two-way ANOVA with Bonferroni post-test). (**C**) Changes of astrocyte size during a one-week period in Ctrl, Rap, KA, Pre-Rap + KA, and KA + Post-Rap groups. Compared to Ctrl, Rap, Pre-Rap + KA, and KA + Post-Rap groups, astrocyte size decreased significantly at 1 wk after seizures in KA group. Both rapamycin pre- and post- treatment reversed the decrease of astrocyte size at 1 wk after seizures (*p < 0.05 compared with KA at 1 wk, by repeated measures two-way ANOVA with Bonferroni post-test). (**D**) Changes of soma size over a one-week period in Ctrl, Rap, KA, Pre-Rap + KA, and KA + Post-Rap groups. There is no significant difference among five groups in soma size at all observed time-points (p > 0.05, by repeated measures two-way ANOVA with Bonferroni post-test). (**E**) Changes of the ratio of soma-to-astrocyte during a one-week period in Ctrl, Rap, KA, Pre-Rap + KA, and KA + Post-Rap groups. The ratio decreased significantly in KA group when compared to Ctrl, Rap, Pre-Rap + KA, and KA + Post-Rap groups at 1 wk after seizures. Both rapamycin pre- and post-treatment reversed the ratio of soma-to-astrocyte at 1 wk after seizures (*p < 0.05 compared to KA at 1 wk by repeated measures two-way ANOVA with Bonferroni post-test).

Our previous studies have demonstrated that rapamycin treatment may attenuate acute dendritic injury caused by KA induced seizures^6^. Therefore, we further tested whether rapamycin treatment would also prevent or rescue seizure-induced astrocyte injury. First of all, rapamycin by itself had no significant effect on astrocytes (Rap group; Fig. 4). To test the effect of rapamycin on seizure-induced astrocytic changes, two different rapamycin treatment paradigms, pre- and post-treatment (see Methods), were performed, which have previously been demonstrated to inhibit KA-induced mTOR activation and associated dendritic injury^6^. No significant changes in astrocyte number were observed in both the rapamycin pre-treatment (Pre-Rap + KA group, Fig. 5) and post-treatment (KA + Post-Rap group, Fig. 6) at all time-points (p > 0.05, by One-way ANOVA with the Tukey’s post-test). Furthermore, no significant differences in astrocyte number were observed between the rapamycin pre- and post-treatment groups at all time-points, compared to Ctrl, Rap alone, and KA groups (p > 0.05, by repeated measures two-way ANOVA with Bonferroni post- test, Fig. 7B).

Importantly, rapamycin pre- and post-treatments were both beneficial in preventing KA seizure-induced astrocyte vacuolization, astrogliosis, and astrocyte morphological changes. Rapamycin had no effect on KA-induced seizure properties themselves (Fig. 1B–E). However, rapamycin significantly prevented acute seizure-induced astrocyte vacuolization immediately after KA induced seizures in the rapamycin pre-treatment group (~4% of astrocytes) as well as in the rapamycin post-treatment group (~34% of astrocytes) (Table 1). Rapamycin pre-treatment was more beneficial than post-treatment in preventing astrocyte vacuolization. In both pre- and post-treatment groups, rapamycin significantly prevented seizure induced astrogliosis and changes in mean fluorescence intensity, astrocyte size and the soma-to-astrocyte ratio over the 1 wk period (Fig. 7A,C,E), with no significant difference between the two rapamycin treatment groups.

## Discussion

In this study, we have documented acute seizure-induced astrocytic injury utilizing *in vivo* cellular imaging methods and implicated the mTOR pathway in mediating these effects. KA-induced seizures caused acute vacuolization of astrocytes within a few hours of seizure onset. While the vacuolization recovered over a one week period, astrogliosis developed, as characterized by morphological changes and decreased astrocyte size. These astrocytic changes were attenuated by rapamycin treatment, supporting the mTOR dependence of this seizure-induced astrocytic injury. While seizure-related astrogliosis has been reported previously in pathological specimens from animal models and epilepsy patients^12–17^, the present study is novel in demonstrating very rapid effects of seizures on astrocytes, as well as acute effects of rapamycin, with live imaging *in vivo*.

Although neurons remain the principal cells mediating epilepsy and its comorbidities, the role of glial cells in regulating epileptogenesis and associated brain injury has become increasingly recognized. In particular, abnormalities in astrocytes have been implicated in promoting epileptogenesis via a diversity of mechanisms, including extracellular ion and neurotransmitter homeostasis, immune and inflammatory processes, and astrocyte-neuronal synaptic signaling^1–3^. Furthermore, astrocytic death, gliosis, or other structural changes in astrocytes are commonly identified in pathological specimens from animal models and patients with epilepsy^12–17^. In contrast to the fixed, static view provided by conventional pathological studies, recent advances with time-lapse imaging of living tissue demonstrate that astrocytes undergo rapid, dynamic structural changes under a variety of physiological and pathological conditions, including neuronal activity-dependent modulation^10, 18–21^. This rapid motility of astrocytes may directly affect neuronal function, such as by modulating the plasticity of synapses and dendritic spines^18, 19^. The present study is significant in demonstrating for the first time that seizures themselves can cause immediate vacuolization of astrocyte *in vivo*, followed by more gradual development of astrogliosis. While the vacuolization was reversible within a few days, the astrogliosis persisted at a week after seizures and presumably represents astrocytic changes that have been documented in previous chronic pathological studies.

Astrocytic vacuolization has primarily been associated with irreversible astrocytic injury or death in a process termed “clasmatodendrosis”. The mechanisms of astrocytic vacuolization remain incompletely defined, but one recent study suggests that F-actin depolymerization accelerates astrocytic vacuolization following pilocarpine induced status epilepticus model via activation of lysosome-derived autophagic mechanisms, and F-actin stabilizer infusion significantly decreases the size and number of the vacuoles in astrocytes^22^. Another study has shown that reduction of MLC1 protein levels causes vacuolization in astrocytes^23^, which may be linked to abnormal cellular ionic and water transport. However, previous studies were based on fixed tissue and documented changes over relatively long time courses, which may miss rapid dynamic changes of astrocytes after acute injury. In this study, utilizing *in vivo* cellular imaging methods, we documented an acute, reversible vacuolization of astrocytes following KA-induced seizures. This vacuolization occurred within a few hours of seizure onset, and reversed over a one-week period. Further studies are needed to determine the functional consequences of this acute vacuolization and its relationship to more classic, chronic astrocytic injury.

In the present study, we also found that reactive astrogliosis developed within several days after KA-induced seizures, as evident by morphological changes and upregulated GFAP expression. Reactive astrogliosis is another important pathological marker in the epileptic brain in animal models and in human patients, and may contribute to epileptogenesis by a variety of mechanisms^1–3^. While hypertrophy of the primary processes occurred following KA-induced seizures, the apparent overall astrocyte size appeared to decrease. Although astrocyte hypertrophy is often thought as a hallmark of astrogliosis, some studies suggest that reactive astrocytes increase the thickness of their main cellular processes but maintain a restricted overall distribution and volume^24^. The acute *in vivo* imaging from the present study helps define the initial time course and evolution of specific morphological changes to astrocytes, reflective of astrogliosis following status epilepticus.

The molecular mechanisms of seizure-induced astrocytic injury are still incompletely understood. The mechanisms driving astrogliosis itself is somewhat controversial, but likely involves altered expression of a number of genes and proteins, leading to both structural and functional changes in astrocytes^25^. The mTOR pathway is a master regulator of a large multitude of proteins and is activated in astrocytes in animal models following seizures and in brain specimens of epilepsy patients^26, 27^. It is plausible that seizure-induced activation of mTOR within astrocytes can upregulate a cascade of downstream proteins that are involved in astrogliosis. A recent study indicates that genetic deletion of mTOR decreases chronic astrogliosis and seizures in the kainate model^28^. Consistent with this study, we find that pharmacological inhibition of mTOR with rapamycin also reduces acute seizure-induced astrocytic injury and astrogliosis. Rapamycin has previously been shown to reduce astrocyte proliferation or astrogliosis in other non-seizure models of central nervous system injury^29, 30^. We have also previously shown that rapamycin can reduce seizure-induced dendritic spine injury^6^. Thus, in addition to the mechanistic implications of these findings, there are potential clinical applications of the use of mTOR inhibitors to prevent seizure-induced brain injury, including both neurons and astrocytes.

Finally, the functional consequences of this acute astrocytic injury are not known, but it is reasonable to hypothesize that seizure-induced astrocytic changes may directly relate to synaptic and dendritic injury following seizures, which have similarly been documented with *in vivo* imaging studies and are reversible with mTOR inhibitors^4–6^. Astrocytes help support and maintain the structural integrity and functionality of synapses, especially of dendritic spines^18, 19^. Collectively, the seizure-induced injury to both astrocytes and neurons could promote progressive epileptogenesis or contribute to cognitive and other neurological deficits in epilepsy patients. Preventative approaches, targeting either seizure-induced astrocyte or neuronal injury, may be effective for alleviating the negative consequences of epilepsy.

## Materials and Methods

### Animals

Two-to-three month old GFAP-GFP transgenic mice expressing enhanced green fluorescent protein (GFP) under a GFAP promoter were used for all experiments^31^. Care and use of animals were approved by the Washington University School of Medicine Animal Studies Committee and followed guidelines from the National Institutes of Health Guide for the Care and Use of Laboratory Animals.

### Surgery

Animal surgeries were performed using aseptic procedures as previously reported^6, 31^. Briefly, mice were anesthetized with isoflurane and held in a custom-made stereotaxic device, which could be mounted to the microscope stage. A heating pad was used to maintain body temperature while under anesthesia. The skull (a round area of ~2 mm in diameter) was carefully thinned to leave about 20 µm of the inner cortical bone. The thinned skull was coated with a layer of cyanoacrylate glue (Krazy Glue, Elmer’s Products) and then covered with a glass coverslip (#1 in thickness, 5 mm in diameter) over the thinned-skull (Supplemental Fig. 1A). Three screw electrodes were placed adjacent to the cranial window to record electroencephalography (EEG). Cyanoacrylate glue and dental cement (SNAP, Parkwell inc) were applied around the edges of the coverslip to stabilize the coverslip and the EEG electrodes to the skull.

### Seizure induction and electroencephalogram recording

After obtaining baseline astrocytes images, the mice were allowed to recover from anesthesia, and EEG data were acquired simultaneously. EEG signals were amplified and filtered (1–100 Hz) using Powerlab PL3508 amplifiers (AD Instruments, Colorado Springs, CO) and digitized (200 Hz) with LabChart (AD Instruments, Colorado Springs, CO). Mice were then injected with KA (Sigma, St. Louis, MO) (20 mg/kg, i.p., KA group) to induce seizures. Control mice received saline injection instead of KA (Ctrl group). Electrographic seizures were recorded by EEG and the cumulative duration of individual seizures was monitored. An individual seizure was defined as a discrete epoch of repetitive spikes or spike-and-wave discharges lasting at least 10 seconds (Fig. 1A). The behavioral correlate of seizures was scored using a modified Racine scale^5^: stage 1 – behavioral arrest with mouth/facial movements, stage 2 – head nodding, stage 3 – forelimb clonus, stage 4 – rearing, stage 5 – rearing and falling, stage 6 – loss of posture, and generalized convulsive activity. Seizure latency, number, score, and total seizure duration during the acute episode of status epilepticus (defined as >30 min of cumulative seizures) were calculated and analyzed, as described previously^6^. After a cumulative 30–45 min duration of electrographic seizures, seizures were terminated by isoflurane anesthesia induction for subsequent post-seizure imaging at 0 and 4 h. The mice were then housed and followed with post-seizure time-lapse imaging for 1 week.

### Rapamycin treatment

Rapamycin (LC Labs, Woburn, MA, USA) was initially dissolved in 100% ethanol (30 mg/mL), stored at −20 °C, and diluted (1:10) in a vehicle solution containing 5% Tween 80, 5% PEG 400 (low-molecular-weight grade of polyethylene glycol), and 4% ethanol immediately before injection. All other chemicals were obtained from Sigma unless indicated otherwise. Two different rapamycin treatment paradigms were performed in this study, based on previous studies demonstrating inhibitory effects of rapamycin on KA seizure-induced mTOR activation^6^. For a pre-treatment study, rapamycin (6 mg/kg, i.p.) was administered 48 and 24 h before KA injection (Pre-Rap + KA group). For a post-treatment study, rapamycin (6 mg/kg, i.p.) was administrated daily for up to 1 week, starting immediately after KA-induced status epilepticus was terminated (KA + Post-Rap group) and mice were followed up for imaging at various times points (0 h defined as immediately after seizure termination and rapamycin administration). Both studies also included two control groups of mice without KA-induced seizures injected with saline (Ctrl) or rapamycin (Rap).

### Two-photon imaging

Baseline images of astrocytes in neocortex were obtained through the thinned skull using a two-photon microscope (LSM 510; Zeiss, Thornwood, NY) with a water immersion objective (Zeiss, 40x, 0.8 numerical aperture (NA), IR-adjusted, Zeiss), as previously described^31^. A Titanium-Sapphire pulsed infrared laser (Coherent, Santa Clara, CA) was used to stimulate GFP at 900 nm. Low-magnification images approximately 50 to 100 μm below the neocortical surface were first obtained to identify regions with GFP positive astrocytes. At higher magnification (3x digital zoom), z-stacks of 6 to 10 images with 1 μm steps were taken. Individual images were acquired at 12 bits with frame averaging (2–4 times). The same excitation laser power and acquisition settings (e.g., detection gain, amplifier offset, amplifier gain) were maintained in individual animals at different time points for direct comparison. Following seizures, the surface vasculature pattern was used to identify the same astrocytes for post-seizure time-lapse imaging at various times (0 h, 4 h, 24 h, 3 days and 1 week) (Supplemental Fig. 1B). Mice were excluded from analysis due to dura damage, excessive bleeding, or obvious opacity of the initial baseline images. All mice included for analysis were successfully followed for one week observation (n = 6 mice per group).

### Post hoc image analysis

Post hoc image analysis was performed using LSM 5 Image Examiner software (Zeiss) and Image J software (NIH) in a blinded fashion to evaluate the changes in fluorescence intensity, astrocyte number, and morphological features of astrocytes (astrocyte size, soma size, soma-astrocyte-ratio, astrocyte vacuolization) over time. A standard area of 150 µm × 150 µm was chosen as the region of interested (ROI) for each mouse, and the same ROI was analyzed at different time points. Astrocyte number was counted in the same ROI at different time points. The fluorescence intensity (GFAP-driven GFP intensity), astrocyte size and soma size at different time points after the seizures were normalized to those at baseline before the seizures in each group. Morphological features of astrocytes were assessed with respect to total astrocyte size (including processes) and soma size, based on area calculations from the projected Z-stacks using ImageJ software. To measure the area of astrocyte soma (excluding branches/fine processes) and total area, lines were drawn as described previously^31^. In addition to surface area measurements, the vacuolization of astrocytes was also recorded. A vacuole was defined as diameter bigger than 0.5 µm and was clearly seen in the astrocyte.

### Statistics

Statistical analysis was performed using GraphPad Prism 5 software. One-way analysis of variance (ANOVA) with Tukey’s multiple comparison was used for parametric comparisons of astrocyte number and soma-to-astrocyte ratio, as well as seizure parameters (latency, number, duration, score). Kruskal-Wallis test with Dunns posttest was used for non-parametric comparisons of mean fluorescence intensity, astrocyte size and soma size. Repeated measures two-way analysis of variance (ANOVA) with Bonferroni post tests for multiple comparisons was used to compare changes in fluorescence intensity, astrocyte number, the size of astrocyte and its soma, and soma-to-astrocyte ratio between different groups. Chi-square test of independence was used to compare the distribution of astrocytes vacuolization between different groups. All data are expressed as mean ± SEM. Statistical significance was defined as P < 0.05.

### Data Availability Statement

The datasets generated during and/or analysed during the current study are available from the corresponding author on request.

## Electronic supplementary material

Supplementary Figure 1
