# Supplementary material for: Rapamycin Attenuates Acute Seizure-induced Astrocyte Injury in Mice in Vivo
Source: Sci Rep. 2017 Jun 6;7:2867. doi: 10.1038/s41598-017-03032-0 (PMC5460181; doi:10.1038/s41598-017-03032-0)

**Supplementary Information for:**

**Rapamycin Attenuates Acute Seizure-induced Astrocyte Injury in Mice in Vivo**

Dongjun Guo^1^, Jia Zou^1^, Michael Wong^1^

^1^ Department of Neurology and the Hope Center for Neurological Disorders, Washington University School of Medicine, St. Louis, MO 63110

**Supplementary Figure**

Supplementary Figure 1. Schematic diagram of the thinned-skull technique for in vivo imaging of astrocytes in GFAP-GFP mice. (A) In the thinned-skull technique, the skull is carefully thinned to the inner cortical bone without directly penetrating the skull to the brain surface. The thinned skull is coated with a layer of cyanoacrylate glue and covered with a glass coverslip over the skull window. (B) A round area of skull (~2 mm in diameter, marked with black circle) over the left somatosensory neocortex is thinned. Right and left frontal “active” EEG and right parietal “reference” electrodes are placed for monitoring of seizure activity. Vasculature images captured after surgery help to ensure repeated observation from the same areas.


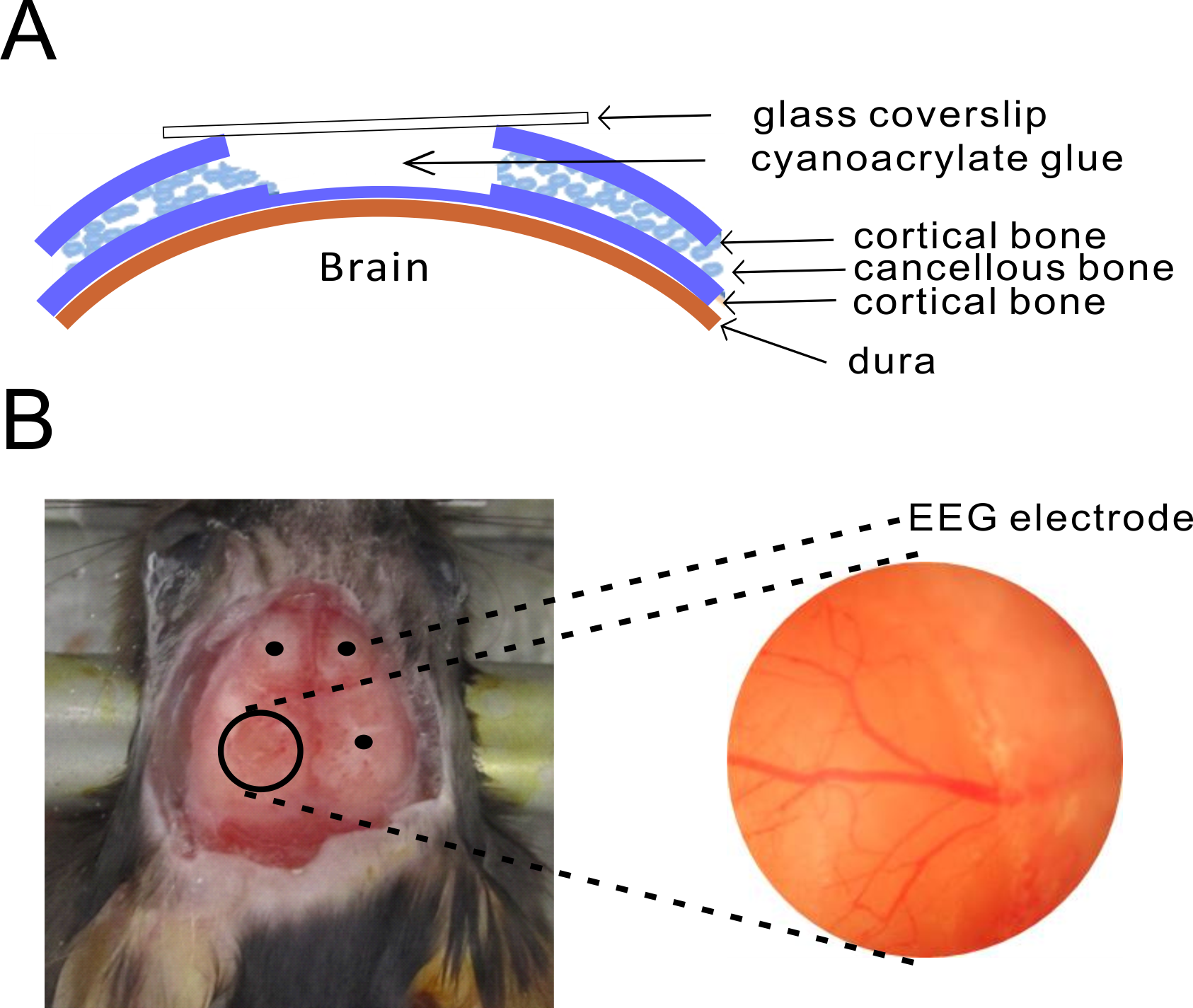

Supplement: Supplementary file 1 — Supplementary Figure 1 [file 41598_2017_3032_MOESM1_ESM.doc]
